# Lumbar and Cervical Concurrent Muscle Activity Analysis During Hamstring Length Testing to Design a Customized Treatment Plan: An Observational Cross-Sectional Study

**DOI:** 10.7759/cureus.62063

**Published:** 2024-06-10

**Authors:** Supriya S, Shantanu Patil, Bagavandas M

**Affiliations:** 1 College of Physiotherapy, SRM Institute of Science and Technology, Chennai, IND; 2 SRM Innovation, Incubation, and Entrepreneurship Centre, SRM Institute of Science and Technology, Chennai, IND; 3 Centre for Statistics, SRM Institute of Science and Technology, Chennai, IND

**Keywords:** customized treatment plan, fundamental study, prevalence, multidisciplinary participants, muscle length testing, muscle work quantification, hamstring length, pressure gauge, concurrent muscle usage, hamstring tightness

## Abstract

Background: Hamstring length plays a significant role in a spectrum of clinical entities, from injury prevention and gait dysfunction to posture correction. Evidence suggests that the prevalence of hamstring tightness (HT)/reduced length is increasing. Despite the number of available tests and treatment protocols, HT is still a functional diagnosis. This study's primary goal is to evaluate concurrent muscle (CM) usage during these testing procedures to design a unique, customized treatment protocol.

Methods: The study was conducted in two stages. In phase 1, Active Straight Leg Raise (ASLR), Active Total Knee Extension (ATKE), and Active Seated Total Knee Extension (ASTKE) were carried out. Next, two pressure gauges (PGs) were placed to align with the natural lumbar and cervical curvatures while testing ASLR and ATKE. After analyzing the results for pressure gauge placement, phase 2 data were collected for tests ASLR and ATKE with PG.

Results: The results of ASLR and ATKE, both with and without PG, indicate a high prevalence rate, whereas the results of ASTKE show no prevalence. Changes in the PG values indicate CM usage. Dichotomization revealed that participants with normal test scores (non-HT group) had increased usage of CM work. Positive and negative changes in PG indicate the involved CM group.

Conclusion(s): In regular practice, most healthcare professionals and fitness trainers prefer ASTKE due to the ease of the testing procedure. Directing co-professionals on their choice of tests is challenging, whereas providing knowledge about CM use paves the way for creating customized treatment plans.

## Introduction

Hamstring muscle length is an important factor for adequate work functioning in normal gait mechanics and injury prevention [1,2]. The prevalence of reduced hamstring muscle length, or hamstring tightness (HT), is highly increasing [3-6]. The standard tests that measure hamstring muscle length focus on the joint angle measurement of the hip and knee, as hamstrings are two joint muscles that cross across both the hip and knee. Some of the active muscle length tests, such as Active Straight Leg Raise (ASLR), Active Total Knee Extension (ATKE), and Active Seated Total Knee Extension (ASTKE), provide information related to the cutoff points that are widely accepted. Any degree less than 80 in ASLR and degrees more than 20 degrees lag in ATKE and ASTKE are considered HT [7]. While these tests do provide sufficient information about HT, it is important to note that there are other muscles, known as concurrent muscles (CMs), that come into action while doing these tests. These CMs are often considered unintentional factors and should be minimized by the tester through careful attention. In addition, the muscular stretching exercises used to enhance the length of the hamstrings primarily target the main muscle group, disregarding the significance of involving the accessory muscle group. This leads to a uniform stretching program for people with decreased muscle length, regardless of their particular variations in muscle activity or engagement. In contrast, this study's research question aims at the quantification of CM usage. This approach to quantifying the CM work while doing a standard test is novel and pioneering, which is necessary to address the prevalence percentage of HT.

Concurrent muscle work is the necessary muscle work that regularly happens with any major functional activity. ASLR, where the hip joint range of motion is measured in supine lying, involves major activation of abdominal muscles and lower back muscles among the group of accessory muscles [8,9]. In other words, individuals undergoing the ASLR test may either flatten or arch the lumbar lordosis, depending on the nature of the individual accessory muscle involvement pattern. Although several groups of muscles get activated, this study focuses on the quantification of CM usage around the lumbar and cervical regions. Two pressure gauge (PG) devices are introduced to align with the natural lumbar and cervical lordosis while performing the tests to monitor the change in pressure difference. The difference between the final and initial readings of PGs, termed the lower back pressure difference (LBPD) and neck pressure difference (NPD), is calculated to quantify CM work. The primary aim of this study is to produce a customized data set for each individual that will serve as both baseline data and a tool to develop a detailed treatment protocol.

This article's pilot data was previously presented as a meeting abstract at the 2023 World Physiotherapy Congress on June 4, 2023.

## Materials and methods

This observational cross-sectional study was conducted at an affiliated institution from July 2022 to July 2023. The inclusion criteria include participants who are willing to perform and capable of performing SLR and TKE without experiencing any pain or discomfort. The study excludes participants who experience knee, low back, or pelvic pain during the examination, have a history of lumbar or lower limb fracture or surgery, have radicular pain, have a visible limb length discrepancy, or are currently pregnant.

The data were collected in two phases: phase 1 pilot study and phase 2 main study. The objectives of the phase 1 pilot study are to identify the prevalence of hamstrings reduced muscle length/HT, to check the difference between ATKE and ASTKE measurements, and to identify the effect of placement of PG devices underneath natural lumbar and cervical curvature in ASLR and ATKE results.

For the phase 1 pilot study, data from 120 therapy student volunteers (21.01 ± 2.39) was collected. First, all three tests, ASLR, ATKE, and STKE, were carried out without PG. Next, two PGs were placed to align with the natural lumbar and cervical curvatures in supine lying while testing ASLR and ATKE. Four physical therapists (other than the primary investigator), in two groups, collected the data. One therapist in each group noted the angles of the tests, while the other recorded the initial and final PG values. All therapists were blinded to each other's data until the completion of the study.

For the phase 2 main study, data from 409 multidisciplinary volunteers (25.76 ± 8.32) was collected. Based on the results of the pilot study, the objective for the second phase was set. Only two tests, ASLR and ATKE with PGs, were carried out to quantify the CM usage. The primary investigator, a physical therapist, performed goniometric measurements, while two trained co-data collectors read the PGs. All tests were done on both sides, the right (R) and left (L) lower extremities.

The sample size for the phase 2 study was calculated based on the prevalence results of the pilot phase 1 study on the local population, with a 97% confidence interval, 3% error, 73.3% prevalence, and 5% precision [10]. All participants were explained regarding the testing procedure with pamphlets and video demonstrations before obtaining their voluntary consent.

The various starting positions for testing procedures are as follows: for ASLR, the participant is supine lying with the contralateral leg maintained on the examination plinth; for ATKE, the tested hip and knee are in a 90-degree fixed position with the contralateral leg maintained in crook lying (supine knee bent) position; and for ASTKE, the participant assumed a seated position on a standard chair, ensuring that both feet were in contact with the ground. For ASLRPG/ATKEPG, in addition to previous start positions, two PGs were placed under the lumbar and cervical spines to align with normal curvatures. Before starting the test, participants were reassured of any discomfort with the placement of PG devices and adjusted accordingly. Lower back pressure and neck pressure are measured at the initial and final stages of the test. Participants were instructed to lift their tested leg up as much as they could comfortably. The subjective response of tolerable movement was considered the end range, at which the test angles were measured. Instruments used include a digital goniometer (two decimal points) and pressure gauge devices (mm Hg). The set cutoff values used to identify the prevalence rate were 80 degrees for ASLR and 20 degrees lag for ATKE.

Statistical analyses

Data were analyzed using SPSS software version 21 (IBM SPSS Statistics, Armonk, NY) and figures with GraphPad Prism version 10.1 (GraphPad Software Inc., La Jolla, CA). Descriptive statistics (mean and standard deviation (SD)) were calculated for age, gender, and body mass index (BMI), along with its subcategory, the measures of angles from each test, and the pressure difference in the lumbar and cervical regions. Additionally, the test results were dichotomized based on the cutoff scores of each test, and the respective pressure difference values were charted. After checking the data for normality, a non-parametric Mann-Whitney U test with a predetermined alpha level of 0.05 was done to check the statistically significant difference present between the right-left sides, above-below cutoff values, and gender differences.

## Results

Participants' demographic characteristics

We included 120 student therapists for the phase 1 study and 409 multidisciplinary participants for the phase 2 study following inclusion/exclusion criteria. Disciplinary information was provided in the Appendices. Demographic details are summarized in Table 1. Two-thirds of the participants were female and one-third were male in both phases of the study. Details of gender- and BMI-classified test results were provided in the Appendices.

**Table 1:** Demographics BMI, body mass index; N, number of participants; n, number in subgroup; SD, standard deviation

Variables	Phase 1 (N = 120) (mean ± SD or n (%))	Phase 2 (N = 409) (mean ± SD or n (%))
Age (mean)	21.01 ± 2.39	25.76 ± 8.32
Gender (female)	76 (63.3%)	276 (67.5%)
Gender (male)	44 (36.7%)	133 (32.5%)
BMI (mean)	18.42 ± 3.92	24.36 ± 4.7
Underweight	71 (59.2%)	42 (10.3%)
Normal	42 (35%)	205 (50.1%)
Overweight	6 (5%)	117 (28.6%)
Obese	1 (0.8%)	45 (11%)

Dichotomization at cutoff values

All test results were dichotomized at cutoff values to understand the pattern of CM usage between the HT group and the non-HT group. The right (R) and left (L) results of ASLR and ASLRPG were tabulated in Table 2 and those of ATKE, ATKEPG, and ASTKE in Table 3. Both dichotomized and combined LBPD and NPD values were provided, along with PG test results. After checking for normality, a non-parametric Mann-Whitney U test was adopted to check the presence of statistically significant differences between tests, sides, and cutoff groups, and p values were tabulated in Table 4.

**Table 2:** ASLR and ASLRPG results ASLR, Active Straight Leg Raise; ASLRPG, Active Straight Leg Raise With Pressure Gauge; HT, hamstring tightness; R, right side; L, left side; LBPD, lower back pressure difference; NPD, neck pressure difference; N, number of participants; n, number in subgroup; SD, standard deviation; NA, not applicable

	Phase 1 (N = 120)			Phase 2 (N = 409)		
	Non-HT group (≥80 degrees) (mean ± SD (n/%))	HT group (<80 degrees) (mean ± SD (n/%))	Combined (mean ± SD)	Non-HT group (≥80 degrees) (mean ± SD (n/%))	HT group (<80 degrees) (mean ± SD (n/%))	Combined (mean ± SD)
ASLR-R	93.7 ± 10.9 (4/3.3%)	54.52 ± 11.05 (116/96.7%)	55.83 ± 13.07	NA		
ASLRPG-R	88.31 ± 6.85 (9/7.5%)	56.83 ± 11.91 (111/92.5%)	59.19 ± 14.27	83.33 ± 3.57 (7/1.7%)	56.28 ± 10.13 (402/98.3%)	56.75 ± 10.64
ASLRPG-R LBPD	37.00 ± 30.49	24.04 ± 26.80	25.01 ± 27.17	84.57 ± 46.14	39.78 ± 28.85	40.55 ± 29.72
ASLRPG-R NPD	0.78 ± 5.65	-0.79 ± 8.17	-0.68 ± 8.00	0.43 ± 6.27	-2.38 ± 8.27	-2.33 ± 8.24
ASLR-L	(0/0%)	48.84 ± 12.16 (120/100%)	48.84 ± 12.16	NA		
ASLRPG-L	99.04 ± 12.80 (13/11.7%)	56.39 ± 12.61 (107/88.3%)	61.02 ± 18.31	83.67 ± 4.92 (6/1.5%)	56.83 ± 9.72 (403/98.5%)	57.23 ± 10.19
ASLRPG-L LBPD	25.69 ± 37.27	15.86 ± 23.10	16.93 ± 24.99	108.67 ± 58.06	39.69 ± 26.27	40.70 ± 28.11
ASLRPG-L NPD	-0.62 ± 4.74	-0.30 ± 4.72	-0.33 ± 4.71	-4.83 ± 8.64	-2.29 ± 6.22	-2.33 ± 6.26

**Table 3:** ATKE/ATKEPG/ASTKE results ATKE, Active Total Knee Extension; ATKEPG, Active Total Knee Extension With Pressure Gauge; ASTKE, Active Seated Total Knee Extension; HT, hamstring tightness; R, right side; L, left side; LBPD, lower back pressure difference; NPD, neck pressure difference; N, number of participants; n, number in subgroup; SD, standard deviation; NA, not applicable

	Phase 1 (N = 120)			Phase 2 (N = 409)		
	Non-HT (≤20 degrees) (mean ± SD (n/%))	HT group (> 20 degrees) (mean ± SD (n/%))	Combined (mean ± SD)	Non-HT (≤20 degrees) (mean ± SD (n/%))	HT group (>20 degrees) (man ± SD (n/%))	Combined (mean ± SD)
ATKE-R	13.25 ± 5.54 (28/23.3%)	41.68 ± 17.00 (92/76.7%)	35.05 ± 19.33	NA		
ATKEPG-R	17.64 ± 2.07 (9/7.5%)	43.62 ± 12.20 (111/92.5%)	41.67 ± 13.61	14.92 ± 4.93 (14/3.4%)	42.48 ± 9.28 (95/96.6%)	41.54 ± 10.45
ATKEPG-R LBPD	13.33 ± 41.40	25.55 ± 24.49	24.63 ± 26.08	55.64 ± 39.71	23.73 ± 22.97	24.82 ± 24.36
ATKEPG-R NPD	3.77 ± 6.89	1.52 ± 10.72	1.69 ± 10.47	0.00 ± 8.41	0.09 ± 9.53	0.09 ± 9.49
ATKE-L	13.6 ± 5.48 (32/26.7%)	48.14 ± 18.68 (88/73.3%)	38.93 ± 22.32	NA		
ATKEPG-L	18.43 ± 1.55 (6/5%)	41.45 ± 11.46 (114/95.0%)	40.30 ± 12.25	14.83 ± 4.36 (13/3.2%)	42.72 ± 9.37 (96/96.8%)	41.84 ± 10.47
ATKEPG-L LBPD	26.67 ± 41.31	24.25 ± 25.60	24.37 ± 26.35	50.62 ± 40.17	22.78 ± 22.34	23.66 ± 23.55
ATKEPG-L NPD	4.17 ± 3.66	2.23 ± 8.86	2.33 ± 8.68	-3.54 ± 5.78	0.08 ± 12.36	-0.19 ± 12.21
ASTKE-R	7.73 ± 4.93 (117/97.5%)	22.63 ± 2.10 (3/2.5%)	8.10 ± 5.40	NA		
ASTKE-L	7.89 ± 5.30 (116/96.7%)	21.85 ± 0.58 (4/3.3%)	8.36 ± 5.78			

**Table 4:** Significant difference evaluation (Mann-Whitney U test p value) (α set at 0.05) * indicates significant values. ASLR, Active Straight Leg Raise; ASLRPG, Active Straight Leg Raise With Pressure Gauge; ATKE, Active Total Knee Extension; ATKEPG, Active Total Knee Extension With Pressure Gauge; ASTKE, Active Seated Total Knee Extension; R, right side; L, left side; LBPD, lower back pressure difference; NPD, neck pressure difference; N, number of participants; n, number in subgroup; SD, standard deviation; NA, not applicable

Variables	Phase 1 (N = 120)			Phase 2 (N = 409)		
	Right		Left	Right		Left
ASLR/ASLRPG	0.045*		0.000*	NA		NA
ATKE/ATKEPG	0.001*		0.133	NA		NA
ATKE/ASTKE	0.000*		0.000*	NA		NA
ASLR R/L	0.000*			NA		
ATKE R/L	0.273			NA		
ASTKE R/L	0.858			NA		
ASLRPG R/L	0.701			0.373		
ATKEPG R/L	0.452			0.790		
ASLRPG LBPD ≥80 degrees/<80 degrees	0.169	0.198		0.002*	0.003*	
ASLRPG NPD ≥80 degrees/<80 degrees	0.451	0.659		0.196	0.222	
ATKEPG LBPD ≤20 degrees/>20 degrees	0.392	0.649		0.000*	0.007*	
ATKEPG NPD ≤20 degrees/>20 degrees	0.803	0.163		0.197	0.006*	

Dichotomizing results showed varied prevalence rates of HT with each test. ASTKE-R and ASTKE-L tests identified 2.5% and 3.3% in phase 1. ASLR-L of phase 1 scored the highest prevalence rate of 100%, with a mean of 48.84 ± 12.16.

**Figure 1:**
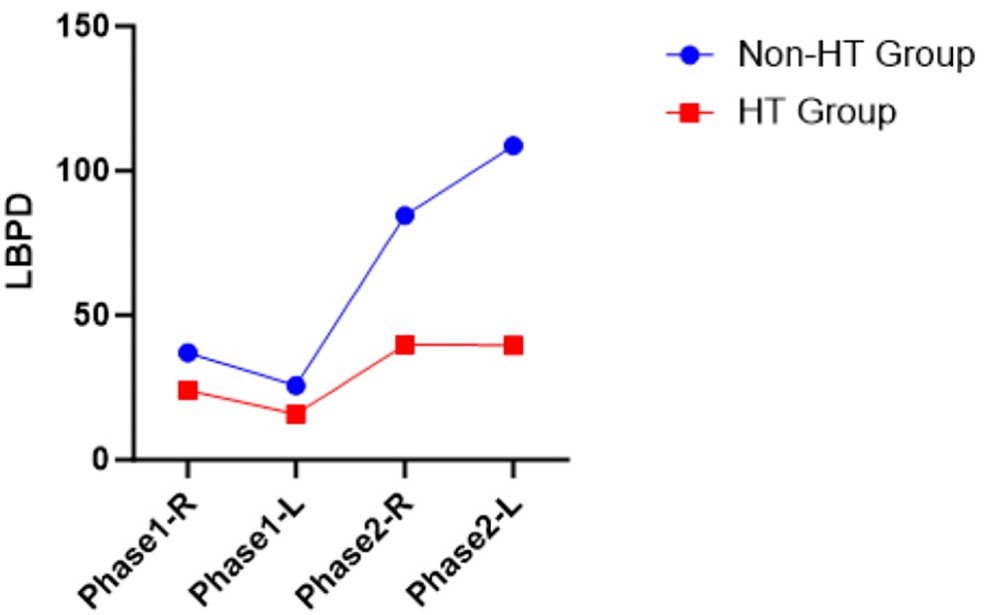
ASLRPG LBPD plotted mean ASLRPG, Active Straight Leg Raise With Pressure Gauge; HT, hamstring tightness; R, right side; L, left side; LBPD, lower back pressure difference

**Figure 2:**
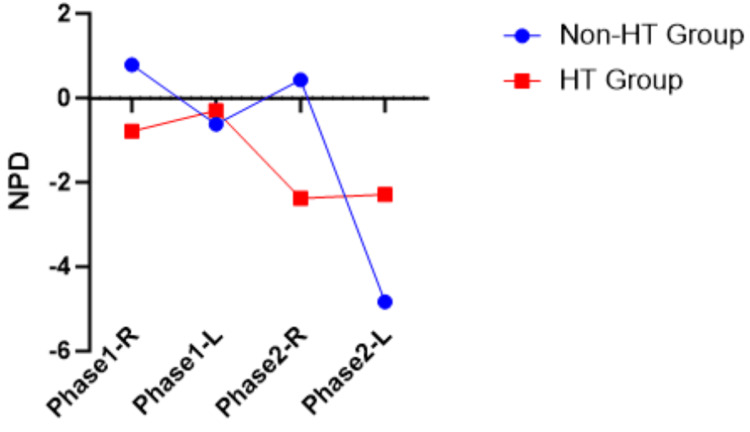
ASLRPG NPD plotted mean ASLRPG, Active Straight Leg Raise With Pressure Gauge; HT, hamstring tightness; R, right side; L, left side; NPD, neck pressure difference

**Figure 3:**
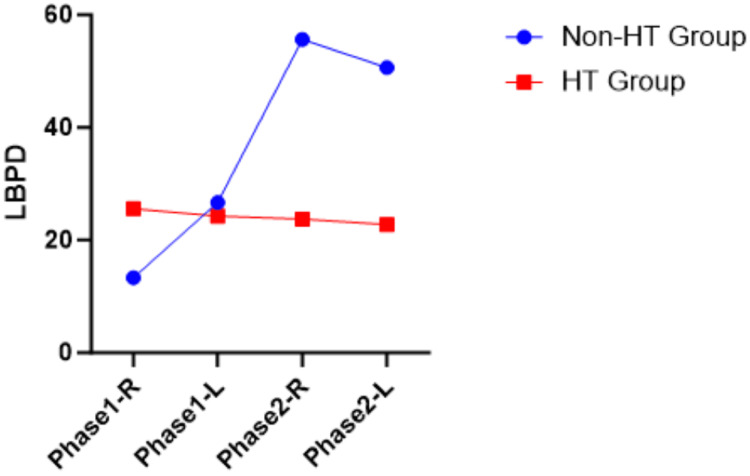
ATKEPG LBPD plotted mean ATKEPG, Active Total Knee Extension With Pressure Gauge; HT, hamstring tightness; R, right side; L, left side; LBPD, lower back pressure difference

**Figure 4:**
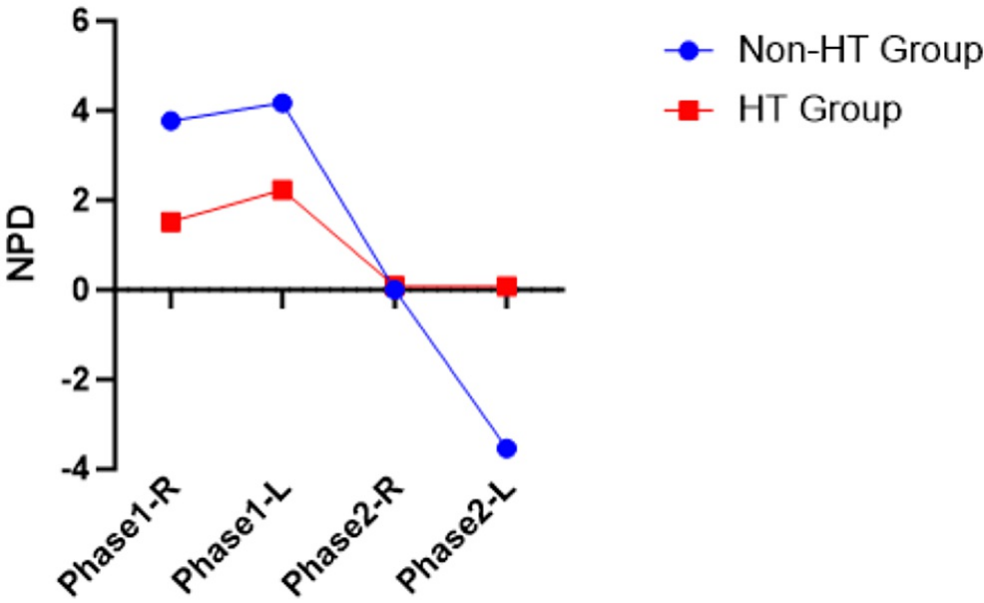
ATKEPG NPD plotted mean ATKEPG, Active Total Knee Extension With Pressure Gauge; HT, hamstring tightness; R, right side; L, left side; NPD, neck pressure difference

LBPD and NPD have both positive and negative values. Visualization of LBPD and NPD between the non-HT group and the HT group for both phases was provided in Figure 1, Figure 2, Figure 3, and Figure 4. The LBPD values of the non-HT group are high compared to those of the HT group in all tests, except phase 1 ATKEPG-R. The variation seen was wider in phase 2 compared with phase 1. Both phase HT group mean NPD of ASLRPG are negative values, whereas the mean NPD values of ATKEPG were positive. The non-HT group NPD values of ASLRPG-R on both phases are increased, whereas the NPD of ASLRPG-L is decreased (more negative values showing increased neck arching) compared to the HT group. The non-HT group NPD of phase 1 TKEPG-R and TKEPG-L were increased compared to the HT group, whereas phase 2 NPD values were decreased. Overall, the NPD variation was noted between sides in ASLRPG and between phases in ATKEPG. A zero mean score of NPD represents the cancellation of positive and negative values.

Placement of PG analysis

**Figure 5:**
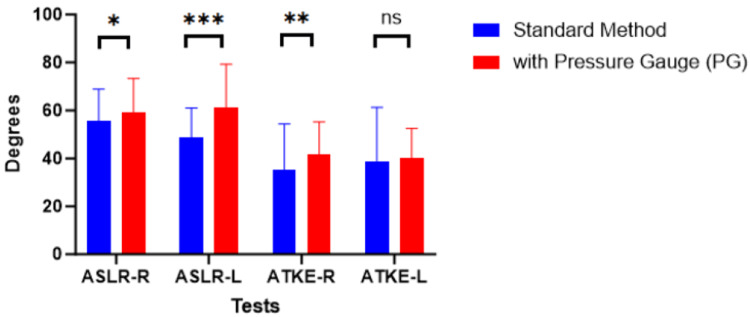
Phase 1 test comparison Bars represent mean ± SD. Significant at *p < 0.05, **p < 0.01, and ***p < 0.001. ASLR, Active Straight Leg Raise; ATKE, Active Total Knee Extension; R, right side; L, left side; PG, pressure gauge; ns, non-significant; SD, standard deviation

Visualization of test results of phase 1 was provided in Figure 5 to analyze the influence of PG introduction. Statistically significant differences were noted on both sides of ASLR (p values from Table 4 show 0.045 for the right side and 0.000 for the left side) and on the R side ATKE (p = 0.001). The HT prevalence rate was reduced with the introduction of PG for both ASLRPG-R and ASLRPG-L, whereas it increased in tests ATKEPG-R and ATKEPG-L. The sum of ranks to analyze the PG device's introduction is tabulated in the Appendices.

Table 5 represents the data of an individual participant. While performing ASLRPG, a positive difference in LBPD and no difference in NPD represents flattening of the lumbar curvatures and no change in the cervical region, and while performing ATKEPG, flattening of the lumbar curvature along with arching of the cervical curvature was noted. The arching or increased cervical curvature is marked by a negative reading in NPD.

**Table 5:** Sample data pattern for an individual ASLRPG, Active Straight Leg Raise With Pressure Gauge; ATKEPG, Active Total Knee Extension With Pressure Gauge; LBPD, lower back pressure difference; NPD, neck pressure difference

Tests	ASLRPG		ATKEPG	
	Right	Left	Right	Left
Degrees	50	54.8	32.5	55.4
LBPD	18	20	52	6
NPD	0	0	-2	-2

## Discussion

Postural asymmetry or movement restrictions lead to compensatory movement patterns in the lumbar spine, and in particular, hamstring flexibility is strongly correlated with hip and lumbar spine motion [11]. While handling manual tasks, HT influenced higher trunk movement amplitudes and restricted pelvic movements [12,13]. In alignment with these findings, several concurrent movements and muscle work accompany testing procedures, such as the hamstring flexibility tests SLR and TKE. Testers focus on reducing this CM work by positioning the contralateral lower extremity in flexed positions [14] or by strapping [3]. Bohannon et al. [15] in their cinematography film analysis study noted that the pelvic rotation began within nine degrees of the beginning of passive SLR and raised awareness about the contribution of pelvic rotation to SLR angle during test interpretation. In this study, we attempted to quantify this CM work in the lumbar and cervical regions using PG devices while conducting the ASLR and ATKE. To our knowledge, this is the first study to utilize the CM work data in designing an intervention plan.

Data were collected in two phases. Phase 1 included 120 therapy student volunteers. Four physical therapy professionals other than the primary investigator collected phase 1 data with complete blinding. Phase 2 included 409 multidisciplinary participants (Appendices). The phase 1 pilot group had two exposures: gold standard tests and testing with PG. The phase 2 group was only exposed to tests with PG. The results revealed a high HT prevalence rate (Table 1 and Table 2) on both sides of tests ASLR, ASLRPG, ATKE, and ATKEPG. In contrast, ASTKE-R and ASTKE-L showed a 2.5% and 3.3% HT prevalence rate, respectively. Similar results were found by Papa and Feland [16].

The third objective of the pilot study was to check the influence of the placement of PG while testing. The higher values of the overall sum of the ranks with ASLRPG compared to ASLR on both sides (Appendices) showed that the placement of PG had a positive influence.

Upon analyzing the results from phase 1, two tests, ASLRPG and ATKEPG, were selected for phase 2. Initial and final readings of lower back and neck PG devices were noted before the start and during the goniometer measurement. The positive change in pressure difference representing the curve's flattening indicates flexor group muscle work and the negative change that arises with the arching of the spinal curvature indicates extensor group muscle work. Further evaluation of pressure difference with dichotomized data above and below the cutoff point revealed that the participants who had normal hamstring length had statistically significant higher values of pressure difference change, indicating the increased usage of CM work to achieve their respective test results. This triggers questions related to variation in CM involvement in stabilizing the pelvis during the testing procedures.

In addition, the phase 1 study participants, students from physiotherapy and occupational therapy, showed an in-depth understanding of the testing procedures, whereas the main study participants from varied groups readily understood the concept after explanation; this reflected different degrees of following the testing procedure, requiring multiple cues and visual demonstration from the tester. We speculate that this might be the reason for the difference in the means of LBPD in ASLRPG between the two phases. The mean average was similar among the ATKEPG tests. Davis et al. [7] suggested adopting the TKE test as the gold standard hamstring length measure until further research. We agree with their results for CM quantification, as the influence of external factors is reduced in the ATKE test.

The main focus of the study is to utilize the data of the CM group in treatment planning. The high standard deviation in the LBPD values shows the uniqueness of each individual in recruiting the core muscle work, showcasing the need for a customized treatment protocol. The data collected from one single participant as in Table 5 serves as the baseline fingerprint of an individual for that particular time. This data pattern serves the therapist in formulating the customized treatment session based on underlying biomechanics. For instance, increased negative values in LBPD raise the need to stretch lumbar extensors before stretching hamstring muscles.

The distribution of test results was the same across the categories of gender (Appendices), whereas there were statistically significant differences present between the categories of body mass index (Appendices), stating the importance of incorporating these results while designing custom treatments.

Limitations

The geographical area of the test conducted is one locality. Conducting the same study in multiple sites may add additional inputs. We have also noticed accompanied rotational component in the hip region and involuntary positioning of the ankle joint in a fixed degree while conducting the tests; we were not able to measure all these details, as this is beyond the scope of the study and due to the complicated nature of collecting these minute details. Future studies focused on adding these additional variables will increase accuracy in the quantification of CM work.

## Conclusions

Most healthcare professionals and fitness trainers prefer ASTKE in regular practice due to the ease of the testing procedure. Overall, the prevalence rate of reduced hamstring length is high. ASLR and ATKE pick up more prevalence percentages than ASTKE. These results will assist healthcare practitioners in their choice of test. Variations in CM work in ASLR and ATKE were evident with PG placements. Directing co-professionals on their choice of tests is challenging, whereas providing knowledge about CM use paves the way for creating relevant treatment plans. Although CM work reduction is possible while conducting the tests, incorporating the levels of use of these muscles enriches the amount of data collected. We found that there is a difference in the recruitment of CM usage and its extent between each participant. Utilizing the CM work data helped us to develop a customized treatment plan in developing baseline data to compare future progress, include multiple muscular factors in addition to joint degree data, and provide a visual plan to share the data with patients, caregivers, and other multidisciplinary professionals.

This study serves as a fundamental study to incorporate multiple variables and use data science in dealing with the complex nature of problems. This way of including more variables in today's data-driven world will pave for more future big data research in the medical sciences. Results are classified into levels of HT for further analysis of clustering and classification.

## Appendices

The details of the participants are presented in Table 6.

**Table 6:** Participants' details N, number of participants; n, number in subgroup; MBBS, Bachelor of Medicine and Bachelor of Surgery

Phases	Participant disciplinary details	n
Phase 1 pilot study (N = 120)	Physiotherapy students	96
	Occupational therapy students	24
Phase 2 main study (N = 409)	Dentist	52
	Nurse	51
	Physiotherapist	55
	Occupational therapist	29
	Speech-language pathologist	37
	Public health professionals	29
	Sports (female volleyball players)	24
	Doctors of pharmacy	54
	MBBS professionals	6
	Optometrist	7
	Nutritionist	2
	Biotechnologist	9
	Statistician	7
	Engineers	8
	General public	39

Details of gender- and BMI-classified test results are shown in Table 7 and Table 8, respectively.

**Table 7:** Gender-classified results (Mann-Whitney U test p value) ASLR, Active Straight Leg Raise; ASLRPG, Active Straight Leg Raise With Pressure Gauge; ATKE, Active Total Knee Extension; ATKEPG, Active Total Knee Extension With Pressure Gauge; ASTKE, Active Seated Total Knee Extension; R, right side; L, left side; LBPD, lower back pressure difference; NPD, neck pressure difference; N, number of participants; SD, standard deviation; NA, not applicable

Variables	Phase 1 pilot study (N = 120)			Phase 2 main study (N = 409)		
	Male (44) (mean ± SD)	Female (76) (mean ± SD)	p value	Male (276) (mean ± SD)	Female (133) (mean ± SD)	p value
ASLR-R	54.68 ± 13.70	56.49 ± 12.74	0.373	NA	NA	NA
ASLR-L	50.15 ± 12.24	48.08 ± 12.13	0.403	NA	NA	NA
ASLRPG-R	59.21 ± 14.36	59.18 ± 14.32	0.842	57.44 ± 11.21	56.41 ± 10.37	0.213
ASLRPG-L	60.37 ± 16.97	61.39 ± 19.14	0.983	56.98 ± 10.27	57.35 ± 10.17	0.683
ATKE-R	35.56 ± 19.73	34.75 ± 19.23	0.926	NA	NA	NA
ATKE-L	36.15 ± 22.13	40.54 ± 22.42	0.298	NA	NA	NA
ATKEPG-R	39.64 ± 13.47	42.85 ± 13.63	0.185	40.64 ± 10.25	41.97 ± 10.53	0.283
ATKEPG-L	38.37 ± 11.91	41.42 ± 12.38	0.143	41.91 ± 10.99	41.80 ± 10.23	0.932
ASTKE-R	8.34 ± 5.43	7.97 ± 5.41	0.651	NA	NA	NA
ASTKE-L	9.12 ± 5.87	7.92 ± 5.73	0.244	NA	NA	NA

**Table 8:** BMI-classified results (Kruskal-Wallis p value) ^a^Phase 1 pilot study ^b^Phase 2 main study ^ab^Combined phase 1 and phase 2 * indicates significant values. BMI, body mass index; ASLR, Active Straight Leg Raise; ASLRPG, Active Straight Leg Raise With Pressure Gauge; ATKE, Active Total Knee Extension; ATKEPG, Active Total Knee Extension With Pressure Gauge; ASTKE, Active Seated Total Knee Extension; R, right side; L, left side; LBPD, lower back pressure difference; NPD, neck pressure difference; SD, standard deviation; -, no data because only one person falls in that category

Variables	BMI category				Kruskal-Wallis test
	Underweight (mean ± SD)	Normal (mean ± SD)	Overweight (mean ± SD)	Obese (mean ± SD)	p value
ASLR-R	57.26 ± 13.27^a^	53.23 ± 12.87^a^	60.48 ± 6.58^a^	-	0.059^a^
ASLR-L	48.39 ± 12.43^a^	49.34 ± 12.43^a^	50.35 ± 9.02^a^	-	0.922^a^
ASLRPG-R	58.03 ± 12.34^a^, 58.99 ± 11.48^b^	58.05 ± 14.63^a^, 56.02 ± 10.52^b^	83.15 ± 14.23^a^, 56.56 ± 10.77^b^	58.46 ± 9.94^b^	0.005^a*^, 0.215^ab^
ASLRPG-L	60.50 ± 17.50^a^, 59.65 ± 8.89^b^	59.03 ± 18.09^a^, 56.32 ± 10.05^b^	83.97 ± 16.66^a^, 57.99 ± 10.46^b^	57.10 ± 11.03^b^	0.013^a*^, 0.090^ab^
ATKE-R	37.11 ± 19.25^a^	31.52 ± 20.02^a^	34.51 ± 15.88^a^	-	0.428^a^
ATKE-L	41.35 ± 22.95^a^	35.09 ± 20.44^a^	35.63 ± 28.66^a^	-	0.330^a^
ATKEPG-R	42.45 ± 13.94^a^, 41.23 ± 8.85^b^	41.32 ± 13.29^a^, 42.13 ± 9.91^b^	35.88 ± 13.47^a^, 41.46 ± 11.96^b^	39.33 ± 10.04^b^	0.741^a^, 0.405^ab^
ATKEPG-L	40.91 ± 12.70^a^, 43.22 ± 10.02^b^	40.43 ± 11.47^a^, 42.95 ± 9.68^b^	34.77 ± 12.20^a^, 40.58 ± 11.68^b^	-38.75 ± 10.36^b^	0.400^a^, 0.043^ab*^
ASTKE-R	8.52 ± 5.51^a^	7.27 ± 5.53^a^	9.17 ± 3.01^a^	-	0.327^a^
ASTKE-L	8.29 ± 5.64^a^	8.71 ± 6.38^a^	6.68 ± 3.48^a^	-	0.958^a^
ASLRPG-R-LBPD	24.47 ± 18.63^a^, 44.29 ± 27.68^b^	23.00 ± 29.73^a^, 38.95 ± 29.34^b^	32.89 ± 30.20^a^, 41.86 ± 30.19^b^	40.91 ± 32.42^b^	0.033^ab*^
ASLRPG-R-NPD	-1.46 ± 5.77^a^, -1.45 ± 5.62^b^	0.29 ± 10.99^a^, -1.22 ± 9.75^b^	1.50 ± 6.75^a^ -3.66 ± 5.89^b^	-4.78 ± 7.14^b^	0.000^ab*^
ASLRPG-L-LBPD	14.79 ± 18.63^a^, 42.50 ± 30.25^b^	20.62 ± 32.27^a^, 39.95 ± 27.40^b^	15.83 ± 35.84^a^, 40.79 ± 28.31^b^	42.22 ± 29.50^b^	0.000^ab*^
ASLRPG-L-NPD	-0.62 ± 4.95^a^, -1.07 ± 4.09^b^	-0.12 ± 4.36^a^, -1.69 ± 6.26^b^	1.16 ± 4.83^a^, -2.96 ± 6.13^b^	-4.76 ± 7.51^b^	0.000^ab*^
ATKEPG-R-LBPD	23.49 ± 21.67^a^, 23.64 ± 19.42^b^	26.14 ± 31.18^a^, 26.18 ± 21.54^b^	20.00 ± 34.06^a^, 21.69 ± 28.90^b^	27.89 ± 27.46^b^	0.541^ab^
ATKEPG-R-NPD	1.92 ± 4.58^a^, 0.81 ± 9.52^b^	1.55 ± 16.66^a^, -0.23 ± 7.02^b^	-0.17 ± 6.01^a^, 1.30 ± 13.60^b^	-2.31 ± 4.88^b^	0.000^ab*^
ATKEPG-L-LBPD	23.40 ± 26.13^a^, 20.50 ± 22.91^b^	27.92 ± 25.85^a^, 25.20 ± 24.11^b^	18.33 ± 30.61^a^, 21.18 ± 22.46^b^	26.09 ± 24.19^b^	0.365^ab^
ATKEPG-L-NPD	3.33 ± 10.15^a^, -3.14 ± 33.01^b^	0.95 ± 5.93^a^, 0.31 ± 5.37^b^	0.67 ± 4.63^a^, 0.28 ± 8.64^b^	-0.91 ± 5.20^b^	0.002^ab*^

Table 9 shows the sum of the ranks of phase 1 test results.

**Table 9:** Sum of the ranks of phase 1 test results BMI, body mass index; ASLR, Active Straight Leg Raise; ASLRPG, Active Straight Leg Raise With Pressure Gauge; ATKE, Active Total Knee Extension; ATKEPG, Active Total Knee Extension With Pressure Gauge; R, right side; L, left side

Tests	Sum of ranks (total sum of all 120 participants results)
ASLR-R	13379.50
ASLRPG-R	15540.50
ASLR-L	11519.00
ASLRPG- L	17401.00
ATKE-R	12693.00
ATKEPG-R	16227.00
ATKE-L	13651.50
ATKEPG-L	15268.50
